# Aurora kinase A promotes hepatic stellate cell activation and liver fibrosis through the Wnt/β-catenin pathway

**DOI:** 10.3389/fonc.2024.1517226

**Published:** 2025-01-06

**Authors:** Guanqi Dai, Junhao Lin, Yuchuan Jiang, Xinhui Liu, Peng Chen, Yixiao Zhang, Zhenghui Song, Xuefen Zhuang, Jinge Cong, Yingchun Li, Xuanjia Hong, Yun Liu, Dong Xiao, Aimin Li, Yue Luo

**Affiliations:** ^1^ Department of Radiotherapy, Southern Medical University Hospital of Integrated Traditional Chinese and Western Medicine, Southern Medical University, Guangzhou, China; ^2^ Cancer Research Institute, School of Basic Medical Science, Southern Medical University, Guangzhou, China; ^3^ Department of Gastroenterology, The Second Affiliated Hospital of Nanchang University, Nanchang, China; ^4^ Department of Hepatobiliary Surgery, The First Affiliated Hospital, Jinan University, Guangzhou, China; ^5^ Department of Endocrinology and Metabolic Diseases, Affiliated Hospital (Clinical College) of Xiangnan University, Chenzhou, China; ^6^ Laboratory Animal Center, Southern Medical University, Guangzhou, China

**Keywords:** Aurora kinase A, hepatic stellate cells, liver fibrosis, MLN8237, Wnt/β-catenin pathway

## Abstract

**Aims:**

Aurora kinase A (AURKA) has been implicated in promoting myeloid and renal fibrosis. This study aimed to investigate the impact and underlying mechanism of AURKA on liver fibrosis and to assess the therapeutic potential of MLN8237, a small-molecule AURKA inhibitor, in preventing liver fibrosis in mice.

**Methods:**

The research used bioinformatics analysis and immunohistochemistry staining on fibrotic liver tissues from human and mouse models to assess AURKA expression. The cellular localization of AURKA was determined through double immunofluorescence staining in human fibrotic liver tissues and primary mouse hepatic stellate cells. RNA interference and AURKA antagonism were used to examine the effects of AURKA on liver fibrosis, while RNA-sequencing, qRT-PCR, and western blotting were employed to elucidate the potential molecular mechanisms of AURKA on hepatic stellate cell activation.

**Results:**

The results showed that AURKA was positively correlated with the progression of liver fibrosis and was predominantly expressed in activated HSCs. Silencing AURKA inhibited HSC activation and proliferation, and induced HSC apoptosis, effects that were similar to those observed with MLN8237 treatment. Additionally, silencing AURKA suppressed the glycogen synthase kinase-3β/β-catenin signaling pathway. Pharmacological inhibition of AURKA phosphorylation also resulted in reduced liver fibrosis *in vivo*.

**Conclusion:**

In conclusion, AURKA may promote HSC activation and liver fibrosis through the Wnt/β-catenin pathway, suggesting its potential as a therapeutic target for liver fibrosis.

## Introduction

Chronic liver disease is a significant global public health concern, affecting over 800 million individuals worldwide and resulting in approximately 2 million deaths annually (1–4). Liver fibrosis, a common pathological process associated with various chronic liver diseases such as chronic viral hepatitis, alcoholic liver disease, non-alcoholic steatohepatitis, and autoimmune hepatitis, is characterized by the accumulation of extracellular matrix proteins (ECM) in the liver (5–8). Without effective intervention, this continuous deposition of ECM proteins can lead to the formation of fibrous scars, distortion of liver structure, and the subsequent development of liver cirrhosis, hepatocellular carcinoma (HCC), and liver failure (9–11). Hepatic stellate cells (HSCs), recognized as pivotal regulatory cells in liver fibrosis, serve as the primary source of ECM protein production and deposition, particularly when they transition to a fibroblast phenotype upon activation (12–14). In the presence of various liver injury factors, signals derived from Kupffer cells, biliary epithelial cells, liver sinusoidal endothelial cells (LESC), platelets, and other cells, along with stimuli, converge on HSCs to promote their activation. Once activated, HSCs transform into myofibroblasts, exhibiting increased proliferation, inflammation, and expression of profibrogenic genes such as alpha-smooth muscle actin (a-SMA), platelet-derived growth factor receptor beta (PDGFRβ), collagen type I alpha 1 chain (COl1A1), and tissue inhibitor of metalloproteinase 1 (TIMP1), thereby exacerbating liver fibrosis (14–17). Therefore, identifying key regulators of HSC activation holds promise for advancing our understanding and future treatment strategies for liver fibrosis.

Aurora kinase A (AURKA) is a serine/threonine protein kinase involved in multiple mitotic events in eukaryotic cells, including centrosome maturation and division, spindle assembly, and localization (18). Recent studies have demonstrated that AURKA plays a significant role in the occurrence, development, and treatment of tumors (19–21). Overexpression of AURKA has been observed in various tumor types, including breast, lung, colorectal, liver, and stomach cancers (22). This overexpression is linked to enhanced tumor proliferation, increased genomic instability, the generation of aneuploid karyotypes, and tumor invasion and metastasis. Furthermore, high levels of AURKA expression are associated with chemotherapy resistance in certain cancers (21). Additionally, AURKA polymorphisms have been correlated with an increased risk of cancer. Given AURKA’s critical role in tumor biology, several AURKA inhibitors, such as MLN8237 (Alisertib) and MLN8054, have progressed to the clinical trial stage (23, 24). The role of AURKA in fibrotic diseases has been less reported, but studies have shown that AURKA plays an important role in alcoholic liver fibrosis, renal fibrosis and myofibrosis (25–27). However, its specific impact on liver fibrosis and hepatic stellate cell (HSC) activation remains relatively unexplored. Here, we aim to investigate the role of AURKA in liver fibrosis and elucidate its underlying mechanism. Our findings suggest that AURKA may enhance HSC activation and liver fibrosis through the Wnt/β-catenin pathway, indicating its potential as a therapeutic target for liver fibrosis.

## Materials and methods

### Chemicals

For *in vitro* experiments, MLN8237 (Selleck Chemicals, Houston, TX, USA) was dissolved in dimethyl sulfoxide (Sigma-Aldrich, St. Louis, MO, USA) and subsequently diluted to the required concentration. For *in vivo* administration, MLN8237 was dissolved in a 0.5% solution of Carboxymethylcellulose sodium (Sigma-Aldrich, St. Louis, MO, USA) at a concentration of 30 mg/kg as previously described (28).

### Small interfering RNA transfection

LX-2 cells were transfected with siRNA, which was designed and synthesized by RiboBio in Guangzhou, China. Transfection was carried out using Lipofectamine 3000 (Invitrogen; Carlsbad; USA) at a concentration of 50nM for either 48 or 72 hours. The specific siRNAs used were siAURKA-1: GAAGAGAGTTATTCATAGA; siAURKA-2: TCTGGCTCTTAAAGTGTTA.

### Human liver samples

All paraffin-embedded tissue samples used in this study were sourced from the Integrated Hospital of Traditional Chinese Medicine at Southern Medical University. Normal liver samples were obtained from individuals who underwent liver resection for benign hepatic diseases, while fibrotic liver samples were obtained from para-carcinoma tissue of hepatocellular carcinoma patients undergoing liver section. All samples were collected with the approval of the Ethics Committee of the Integrated Hospital of Traditional Chinese Medicine, Southern Medical University, and all patients signed informed consent.

### Liver fibrosis models

Mouse models of liver fibrosis were established following previously reported methods. Briefly, male C57BL6J mice aged 4-5 weeks were obtained from Southern Medical University (Guangzhou, China). The CCl_4_-induced liver fibrosis model was created by administering a 40% solution of CCl_4_ in olive oil via oral gavage for either 4 or 8 weeks (29, 30), while the bile duct ligation liver fibrosis model was created through the surgical isolation and ligation of the common bile duct (31). *In vivo* administration of MLN8237 involved treating 4- to 5-week-old male C57BL6J mice with CCl_4_ for 4 weeks, followed by gavage with 30 mg/kg MLN8237 or an equal volume of vehicle reagent (CMC-na) five times a week from 5 to 8 weeks. Mice were euthanized 72 hours after the final treatment, and their liver and serum samples were collected for further biochemical and pathological analyses. The mice were housed in a pathogen-free laminar flow environment, maintained under a 12-hour light-dark cycle at 22°C to 25°C, with free access to standard laboratory mouse food and water. The animal experiments were approved by the Southern Medical University Bioethics Committee and conducted in accordance with established guidelines.

### Primary cell isolation and identification

Primary mouse HSCs isolation was performed by enzymatic digestion followed by density gradient centrifugation. The isolated primary cells were then resuspended in a high-glucose DMEM medium supplemented with 10% FBS and 1% penicillin-streptomycin in 24-well culture plates to induce culture-activated HSCs as described previously (32).

### Cell cultures

The human HSC cell line (LX-2) was obtained from the Chinese Academy of Sciences Cell Bank in Shanghai, China. The cells were grown in high glucose Dulbecco’s Modified Eagle Medium (Gibco, NY, USA) with 10% fetal bovine serum (Gibco, NY, USA), 100 U/ml penicillin, and 100 U/ml streptomycin. The culture was incubated at 37°C in a 5% CO_2_ atmosphere.

### EdU proliferation assay

The Cell-Light Edu Apollo 567 *in vitro* Imaging Kit (RiboBio, Guangzhou, China) was utilized to assess cell proliferation. Following transfection with AURKA siRNA for 48 hours, 10,000 cells were seeded into 96-well microplates and subjected to EdU staining according to the manufacturer’s protocol. Cells were incubated with EdU for 0.5 h, followed by Hoechst 33342 and EdU staining. Five random microscopic fields were examined for each sample to count the cells.

### Cell cycle and cell apoptosis assays

The cell cycle was analyzed using a cell cycle detection kit (MultiSciences, Hangzhou, China) following the provided instructions. DNA content was assessed with FACS caliber flow cytometry (BD Biosciences, New Jersey, USA), and the percentages of cells in each cell cycle phase were determined using ModFit LT V4.1.7 software. Cell apoptosis was evaluated using the FITC Annexin V Apoptosis Detection Kit (BD Biosciences, New Jersey, USA) as per the manufacturer’s guidelines. The apoptotic rate was determined using FACS caliber flow cytometry (BD Biosciences, New Jersey, USA), focusing on early apoptotic cells.

### Western blot

Cells were collected and lysed using a RIPA solution to extract protein samples. Equal amounts of protein were then separated by electrophoresis on a 10% sodium dodecyl sulfate/polyacrylamide gel and subsequently transferred to nitrocellulose membranes. Subsequently, the membranes were incubated overnight at 4°C with a primary antibody according to the manufacturer’s instructions. Immunoreactive proteins were detected using an enhanced chemiluminescence substrate. The primary antibodies utilized for immunohistochemistry staining and Western blot are detailed in **Supplementary Table 1**.

### Real-time quantitative PCR assay

Total RNA from LX-2 cells was isolated using the standard Trizol method (TakaRa Bio Inc., Shiga). Following the manufacturer’s protocol, 1μg of mRNA was utilized to synthesize cDNA with the cDNA Synthesis Kit (TakaRa Bio Inc., Shiga). The qRT-PCR assay was conducted with SYBR Premix Ex Taq II (TakaRa Bio Inc., Shiga) on a Light Cycler 480 II (Roche) as per the manufacturer’s instructions. Samples were analyzed in triplicates and normalized to β-Actin expression using the 2-ΔΔCt method. The specific primers employed are detailed in **Supplementary Table 2**.

### Histology immunohistochemistry and immunofluorescence

Formalin-fixed, paraffin-coated liver tissues were cut into 4 µm sections and stained with Hematoxylin-eosin, Sirius red, and immunohistochemistry techniques. Hematoxylin and eosin staining was performed with Harris’s Hematoxylin (JLM-114, Jielibio) and Eosin Y (BA4040, Bessobio) according to the standard protocol. Sirius red staining was performed using (9046; Chondrex, Sirius Red/Fast Green Collagen Staining Kit) according the manufacturer’s instruction. The degree of liver fibrosis was evaluated blindly by two pathologists according to the Ishak fibrosis score (33). For immunohistochemistry, liver sections were repaired by high pressure in EDTA solution and subsequently stained with the primary antibodies AURKA (1/200; Biorbyt; orb224015), α-SMA (1/1000; Abcam; 19245S) according to the manufacturer’s protocol, Primary antibody incubation was performed at 4°C overnight, followed by incubation with the secondary antibody for 1 hour at room temperature. Staining results were evaluated by integrating the staining intensity and quantity scores, as previously reported (34). For dual immunofluorescence staining, sections fixed with ice-cold methanol were co-stained with AURKA (1/100; Biorbyt; orb224015) and α-SMA (1/500; sigma; C6198), utilizing the appropriate secondary antibodies labeled with Alexa Fluor 488 or Alexa Fluor 594, following the manufacturer’s instructions. Nuclei were stained with DAPI.

### Cell immunofluorescence

In the dual immunofluorescence staining procedure, cells were fixed using ice-cold methanol and co-stained with AURKA (1/100; Biorbyt; orb224015) and α-SMA (1/500; Abcam; ab7817). Subsequently, the cells were incubated with secondary antibodies labeled with Alexa Fluor 488 or Alexa Fluor 549 as per the manufacturer’s guidelines. DAPI was used to stain the cell nuclei.

### Liver function test

Serum was separated from whole blood by centrifugation at 3000 rpm for 10 minutes and stored at -80°C for liver function tests. These tests included alkaline phosphatase (ALP), alanine aminotransferase (ALT), aspartate aminotransferase (AST), total bilirubin (TBIL), albumin (Alb), and glucose (Glu). The biochemical markers were measured using the Roche Cobas 6000 Analyzer (Roche, Basel, Switzerland).

### RNA sequencing

The sample processing, sequencing, and data analysis were consistent with previous reports (35). The Kyoto Encyclopedia of Genes and Genomes (KEGG) pathway analysis was conducted using DAVID (version 6.8, https://david.ncifcrf.gov/) (36). Gene Set Enrichment Analysis was carried out using the Gene Set Enrichment Analysis (GSEA) software, which was downloaded from https://www.gsea-msigdb.org/gsea/index.jsp (37). The protein-protein interaction (PPI) network was analyzed and constructed using the STRING database (http://string-db.org/).

### Statistical analyses

SPSS 21.0 software (IBM, Armonk, NY, USA) was employed for data analysis. The student’s t-test was applied to compare differences between subgroups of measurement data that followed a normal distribution, while the Mann-Whitney U test was utilized for non-normally distributed data. The two-tailed Pearson correlation test was used to determine the correlation coefficient between the two sets of measurement data. Results are presented as the mean ± standard deviation (SD) of at least three replicates. A significance level of P < 0.05 was considered statistically significant.

## Results

### Upregulated AURKA in liver fibrosis is associated with HSC activation

**Figure 1:**
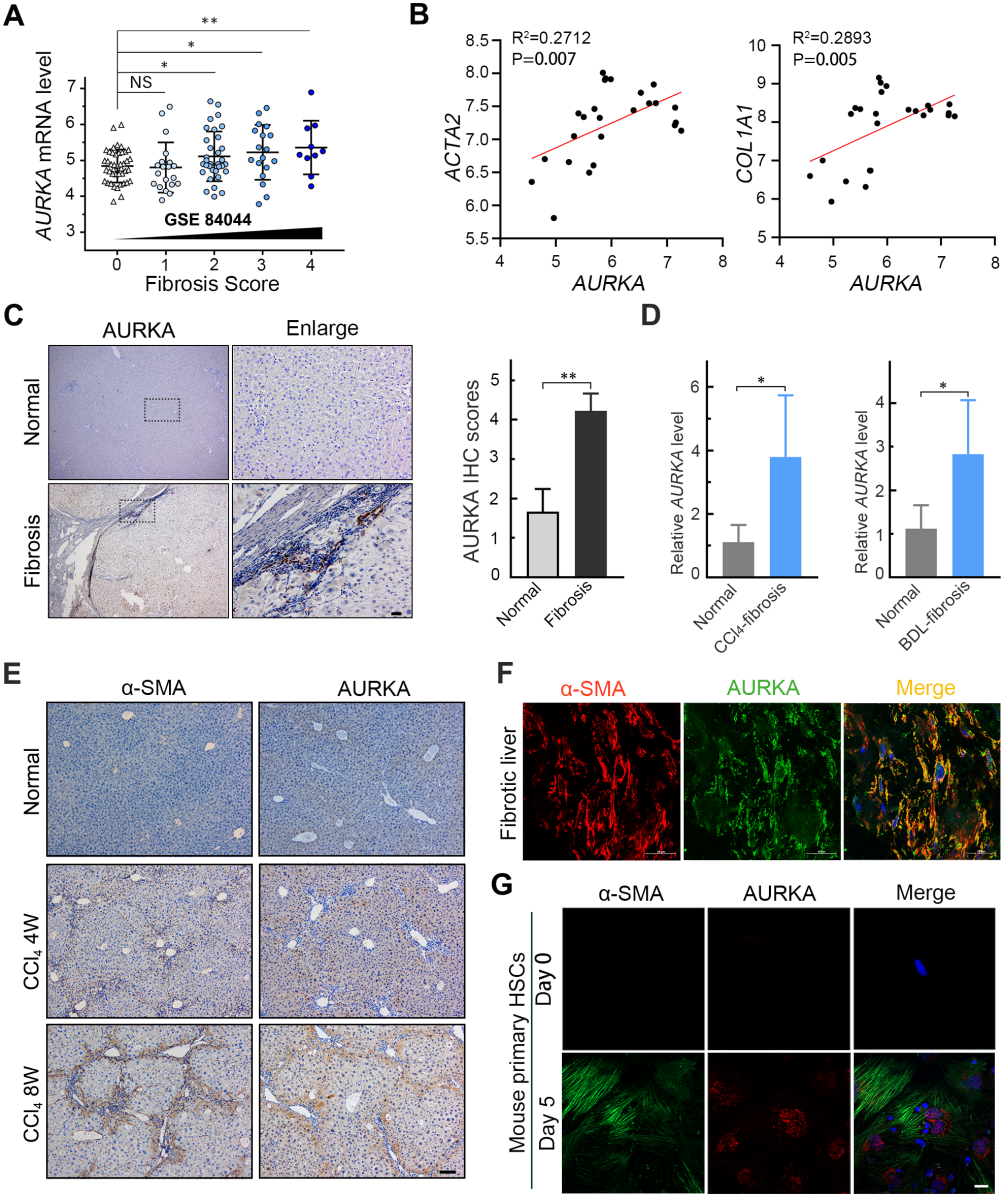
Upregulated AURKA in liver fibrosis is associated with HSC activation. **(A)** Relative AURKA mRNA expression in human liver fibrosis samples obtained from the GEO dataset (GSE84044). **(B)** A positive correlation was observed between the mRNA levels of AURKA and those of ACTA2 and COL1A1 in human fibrotic liver samples from the GEO dataset (GSE38941). **(C)** Representative images of AURKA immunohistochemistry staining in human liver fibrosis/cirrhosis (n =9) and benign hepatic diseases (n =3) and the results of semi-quantitative immunohistochemical analysis. Scale bars, 25μm. **(D)** Relative mRNA expression of AURKA in CCl_4_ (n =5) and Bile duct ligation (n =7) induced liver fibrosis mouse model. **(E)** Representative images of α-SMA and AURKA staining in liver sections of mice treated with CCl_4_ and control group for 4 or 8 weeks. Scale bars, 100μm. **(F)** Represent double immunofluorescence images of α-SMA (red) and AURKA (green) in fibrotic liver tiusse. Scale bars, 25μm. **(G)** Double-immunofluorescence staining of AURKA and α-SMA in mouse primary HSCs cultured for 0 and 5 days *in vitro*. Scale bars, 100μm. Data presented as means ± SD. NS, not significant; *P < 0.05; **P < 0.01.

Our study revealed that AURKA expression increases with the progression of liver fibrosis (**Figure 1A**) and is positively correlated with ACTA2, a marker of activated HSCs (**Figure 1B**), as well as the fibrogenic genes COL1A1 and TIMP1 (**Figure 1B**; **Supplementary Figure S1**), based on publicly available gene expression datasets from liver fibrosis patients. Additionally, immunohistochemistry staining demonstrated a significant upregulation of AURKA in human liver fibrosis and cirrhosis tissues compared to normal liver tissues (**Figure 1C**). Consistent with these findings, increased AURKA mRNA expression was observed murine fibrotic livers induced by CCl_4_ and bile duct ligation, in comparation to normal liver tissues (**Figure 1D**). Interestingly, AURKA-positive cells were predominantly localized in the fibrous compartment of the fibrotic liver (**Figures 1C, E**). Immunofluorescence double staining in human fibrotic liver confirmed the high expression of AURKA in activated HSCs (**Figure 1F**). Our findings from primary mouse HSC differentiation experiments showed a notable increase in both AURKA and α-SMA at day 5 compared to day 0 (**Figure 1G**), suggesting a potential role for AURKA in HSC activation during liver fibrosis.

### Silencing AURKA inhibited HSC activation, proliferation, and induced HSC apoptosis

**Figure 2:**
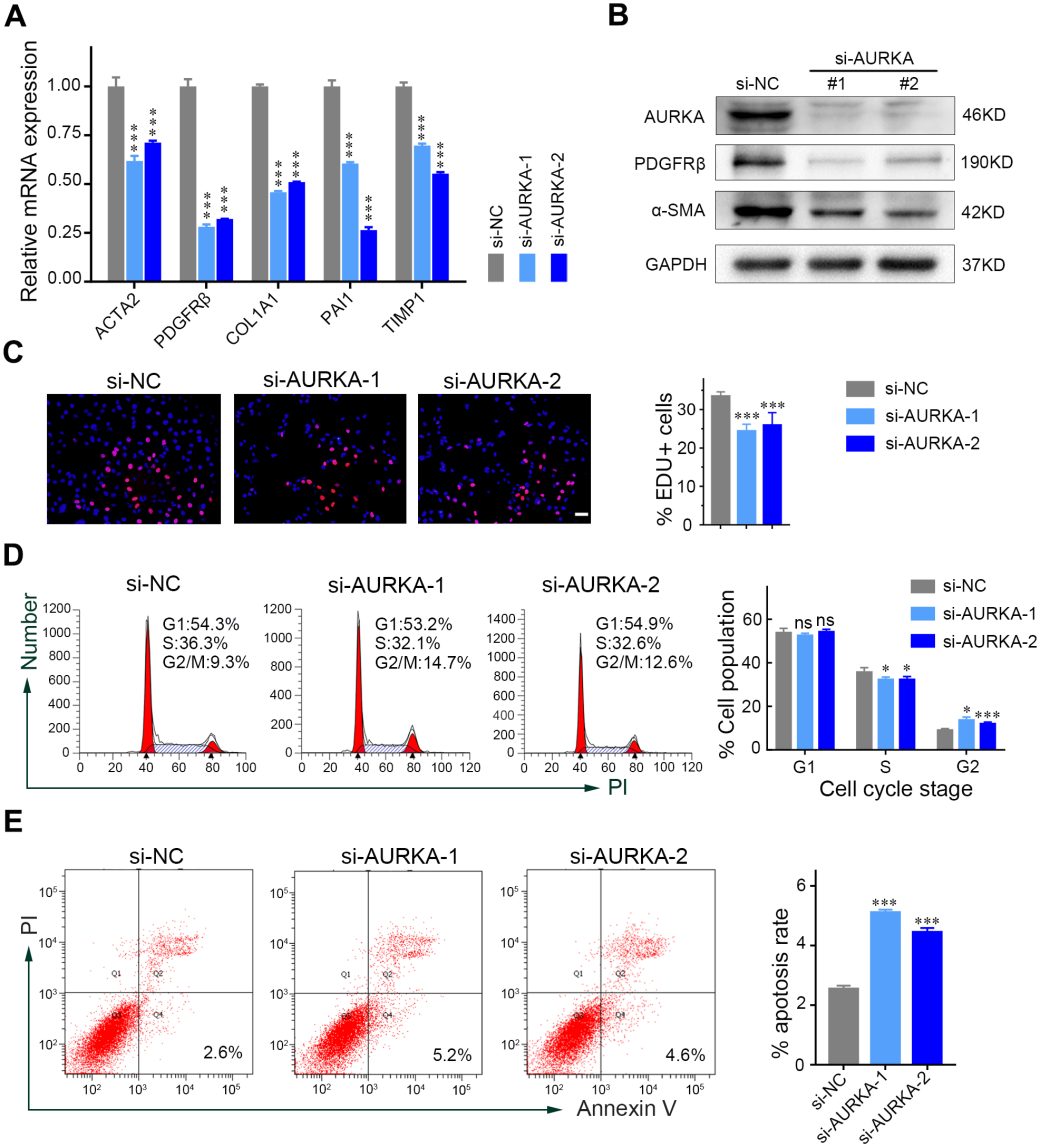
AURKA silence inhibits HSC activation, proliferation, and induces HSC apoptosis. **(A)** Relative mRNA expression levels of HSC activation and fibrogenic genes in LX-2 cells transfected with AURKA siRNAs or control siRNA. **(B)** Immunoblots of the indicated proteins treated with AURKA siRNAs or control siRNA. **(C)** The proliferation level of LX-2 cells following AURKA siRNA treatment was detected by EdU assay. Scale bars, 50μm. **(D)** Cell cycle analysis of LX-2 cells following transfection with AURKA siRNA conducted by flow cytometry. **(E)** The percentage of apoptotic LX-2 cells after incubation with AURKA siRNA. Data presented as means ± SD. NS, not significant; *P < 0.05; ***P < 0.001.

To further elucidate the role of AURKA in HSCs, siRNA was employed to silence AURKA expression in LX-2 cells. The study revealed that knocking down AURKA led to a significant decrease in the mRNA expression of HSC activation markers (ACTA2, PDGFRβ) and profibrogenic genes (COL1A1, PAI1, TIMP1) (**Figure 2A**). Moreover, the protein levels of α-SMA and PDGFRβ were notably reduced in LX-2 cells following AURKA inhibition (**Figure 2B**), highlighting AURKA’s role in promoting HSC activation. In addition, AURKA knockdown resulted in a marked decrease in LX-2 cell proliferation (**Figure 2C**). Consistent with these, flow cytometry analysis showed that the inhibition of AURKA expression induced G2/M phase arrests and apoptosis in LX-2 cells (**Figures 2D, E**).

### AURKA antagonism displayed anti-fibrotic activity *in vitro*

**Figure 3:**
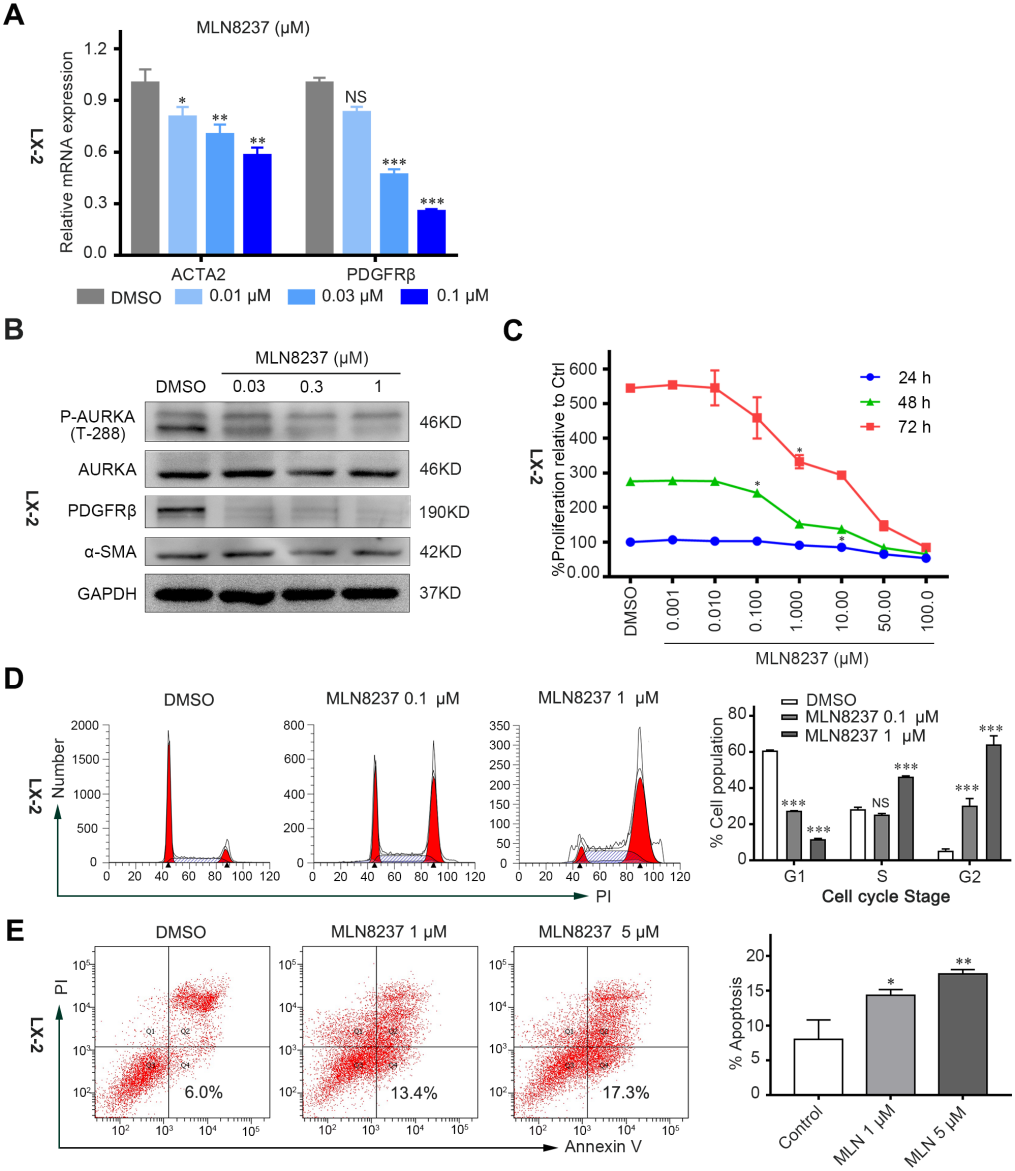
AURKA antagonism has anti-fibrotic activity *in vitro*. **(A)** Relative mRNA expression levels of indicated mRNA in LX-2 cells after being treated with different concentrations of MLN8237 for 48 hours. **(B)** Immunoblots of indicated proteins in LX-2 cells after MLN8237 treatment for 48 hours. **(C)** MLN8237 inhibited cell proliferation of LX-2 cells in a dose and time-dependent manner. **(D)** Cell cycle analysis of LX-2 cells after MLN8237 administration for 48 hours by Flow cytometry. **(E)** The percentage of apoptotic LX-2 cells after 48 hours of MLN8237 treatment. Data presented are means ± SD. NS, not significant; *P < 0.05; **P < 0.01; ***P < 0.001.

To further clarify the anti-fibrotic activity of AURKA antagonism *in vitro*, MLN8237, a specific AURKA inhibitor, was utilized to inhibit AURKA phosphorylation in LX-2 cells. The results demonstrated that MLN8237 effectively suppressed AURKA kinase activity (**Figure 3B**) and downregulated the mRNA and protein levels of α-SMA and PDGFRβ in LX-2 cells (**Figures 3A, B**). Furthermore, MLN8237 treatment inhibited LX-2 cell proliferation in a dose-dependent manner (**Figure 3C**). Additionally, fluorescence-activated cell sorting analysis revealed that MLN8237 induced G 2/M phase cell cycle arrest (**Figure 3D**) and apoptosis in LX-2 cells (**Figure 3E**). In summary, these findings highlight the anti-fibrotic potential of MLN8237 *in vitro*.

### AURKA may activate HSCs by stimulating the Wnt/β-catenin signaling pathway

**Figure 4:**
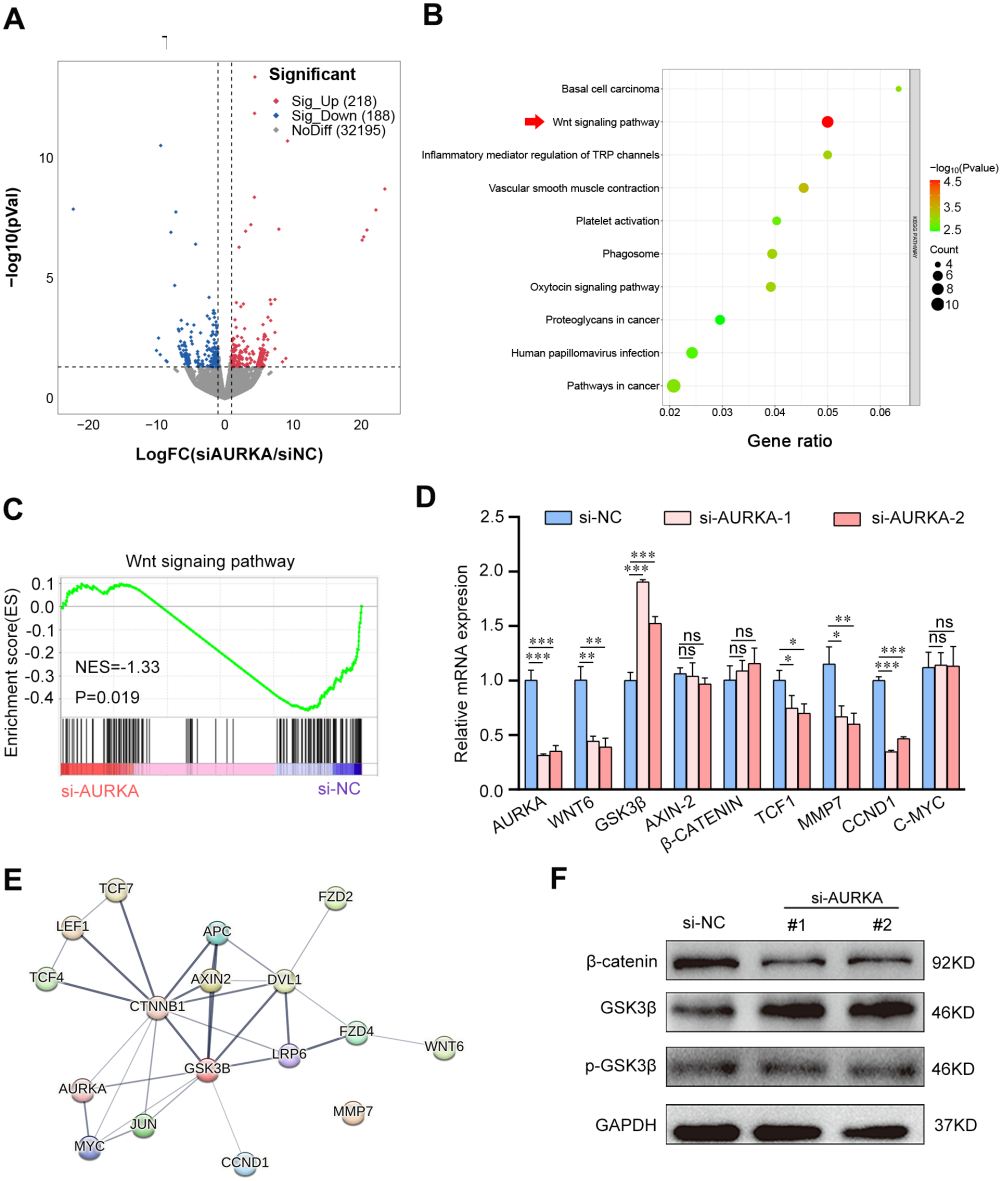
AURKA may activate HSCs by activating the Wnt/β-catenin signaling pathway. **(A)** The volcano plot of RNA-seq analysis in LX-2 cells transfected with either AURKA siRNA or negative control siRNA (n=3). **(B)** The KEGG pathway enrichment analysis of differentially express genes (DEGs) in LX-2 cells transfected with AURKA siRNA compared to the control siRNA group. **(C)** The GSEA plot of enrichment in the ‘Wnt signing pathway’’ in AURKA knockdown LX-2 cells (n=3) utilizing the C2.CP-KEGG MSigDB database. **(D)** Relative mRNA expression levels of Wnt/β-catenin pathway in LX-2 cells transfected with either AURKA siRNA and control siRNA. **(E)** PPI network analysis of AURKA and critical member of the Wnt/β-catenin pathway. **(F)** Expression levels of the indicated protein from LX-2 cells transfected with either AURKA or control siRNA. Data presented are means ± SD. NS, not significant; * *P* < 0.05; **P < 0.01; ***P < 0.001.

To investigate the impact of AURKA on HSCs, RNA sequencing analysis was conducted on LX-2 cells treated with either AURKA siRNA or control siRNA. A total of 406 genes exhibited differential expression following the silencing of AURKA in LX-2 cells. (*P*<0.05, |log2FoldChange|>1) (**Figure 4A**; **Supplementary Table 3**). Subsequent KEGG pathway enrichment analysis revealed significant enrichment of the Wnt signaling pathway (**Figure 4B**). Consistently, GSEA further confirmed the inhibition of the Wnt signaling pathway in LX-2 cells following AURKA interference (**Figure 4C**; **Supplementary Table 4**). The Wnt/β-catenin signaling pathway plays a crucial role in HSC activation and the development of liver fibrosis (38). Thus, RT-qPCR was employed to evaluate the mRNA expression levels of components within Wnt/β-catenin pathway in AURKA knockdown LX-2 cells. The results demonstrated a significant inhibition of the Wnt/β-catenin pathway in these cells, characterized by upregulated GSK3β and downregulated expression of WNT6, TCF1, CCND1, and MMP7 (**Figure 4D**). Further PPI analysis indicated interactions among AURKA, GSK3β, and β-catenin within the Wnt/β-catenin signaling pathway (**Figure 4E**). Western blot validation confirmed that silencing AURKA resulted in increased GSK3β and decreased β-catenin protein expression (**Figure 4F**). These findings suggest that AURKA promotes HSC activation through the Wnt/β-catenin signaling pathway.

### MLN8237 attenuates CCl_4_-induced liver fibrosis *in vivo*

**Figure 5:**
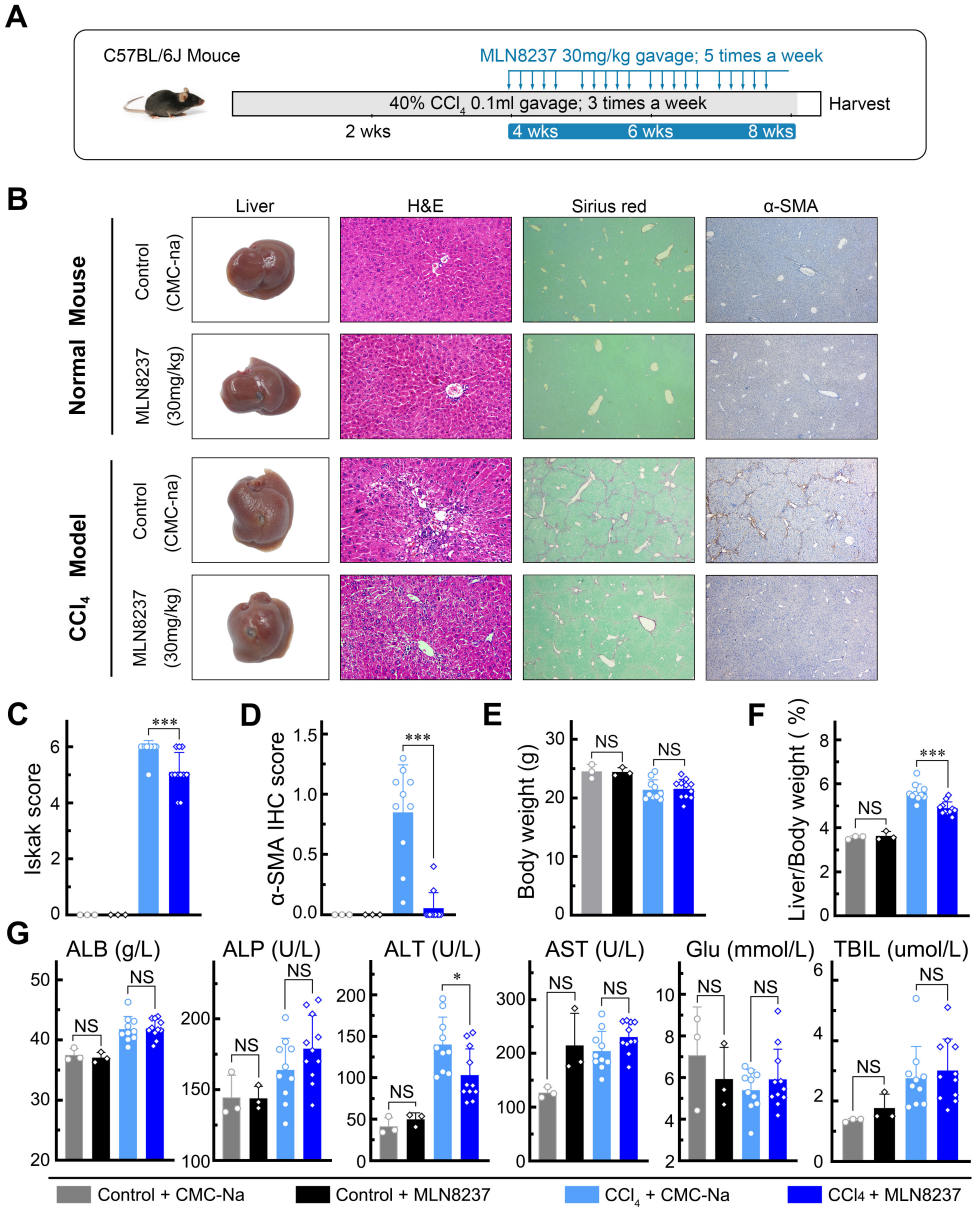
MLN8237 attenuates CCl_4_-induced liver fibrosis *in vivo*. **(A)** The Schematic of the experimental design for MLN8237 treatment in a CCl_4_-induced fibrosis model in mice. **(B)** Representative images of mouse livers stained with H&E, Sirius red, and α-SMA antibodies. **(C)** Ishak fibrosis score, **(D)** α-SMA IHC score, **(E)** body weight, **(F)** liver/body weight ratio, and **(G)** Liver function of mice in each group of mice. Data presented as means ± SD. NS, not significant; *P < 0.05; ***P< 0.001.

To evaluate the efficacy and safety of MLN8237 in the treatment of fibrosis, a CCl_4_-induced liver fibrosis mouse model was established (**Figure 5A**). Treatment with MLN8237 resulted in a significant reduction in cellular damage and a reversal of liver fibrosis compared to the control group, as evidenced by H&E and Sirius red staining (**Figures 5B–D**). Furthermore, MLN8237 effectively suppressed the activation of hepatic stellate cells in the CCl_4_-induced fibrotic liver (**Figures 5B, D**). The liver to body weight ratio was notably lower in the CCl_4_ + MLN8237 group than in the CCl_4_ + CMC-na group (**Figure 5F**). Serological analysis revealed a significant decrease in ALT activity with MLN8237 treatment compared to the control group (**Figure 5G**). Importantly, no significant differences were observed in various parameters between the Normal + MLN8237 group and the Normal + CMC-na group, suggesting a favorable safety profile for MLN8237 *in vivo* (**Figures 5C–G**; **Supplementary Figure S1**). These findings demonstrate that MLN8237 exerts a significant anti-fibrotic effect with good tolerability and safety *in vivo*.

## Discussion

Liver cirrhosis and fibrosis represent significant global health challenges with limited effective treatment options (4, 39–44). The activation of hepatic stellate cells (HSCs) plays a critical role in developing liver cirrhosis and fibrosis, yet the precise mechanism driving this activation and the subsequent transition to a fibrogenic phenotype remains incompletely understood. This study indicates that targeting AURKA could offer a promising therapeutic strategy for liver fibrosis, providing good tolerability and safety in the absence of approved treatments.

Previous research has established the significance of HSC activation and apoptosis in the progression of liver fibrosis (45–48). However, few studies have investigated the relationship between AURKA and liver fibrosis. Zhenni Liu et al. demonstrated that AURKA ubiquitination, mediated by CD73/NT5E, regulates alcohol-related liver fibrosis by influencing HSC senescence. This study reveals that AURKA expression is significantly upregulated in the liver tissues of patients with alcohol-related liver fibrosis, as well as in liver tissues from mouse models of acetaldehyde and carbon tetrachloride-induced liver fibrosis, and in HSCs stimulated by acetaldehyde. The AURKA-specific inhibitor MLN8237 can significantly inhibit the activation of HSCs both *in vivo* and *in vitro*, promoting their senescence and consequently inhibiting the progression of alcoholic liver fibrosis. However, the mechanisms by which AURKA promotes HSC activation and senescence have not been further explored in this study (26). Our findings suggest that AURKA may contribute to the progression of liver fibrosis by enhancing HSC activation and proliferation while inhibiting HSC apoptosis. Treatment with the AURKA inhibitor MLN8237 effectively reduced hepatic fibrosis and HSC activation, thereby improving liver function in a CCl_4_-induced mouse model of liver fibrosis, without significant adverse effects. Nevertheless, further investigations using various animal models that represent diverse etiologies of liver fibrosis are warranted to validate these findings.

Human protein kinases encompass a diverse group of enzymes which can be categorized into serine, threonine, tyrosine, and bispecific protein kinases (49, 50). Research indicates that various protein kinases play a crucial role in the progression of liver fibrosis (34, 51, 52). AURKA, a serine/threonine protein kinase, is pivotal in multiple aspects of cell mitosis (53, 54). It governs critical events such as centrosome maturation, cell division, spindle assembly, and localization during mitosis (53, 54). In the late stage of mitotic G2 in human cells, AURKA gradually accumulate in the centrosome, accompanied by a significant increase in its kinase activity and expression level. The phosphorylation of AURKA activates PLK1, which in turn activates CDK1, a necessary process for cells transitioning from the G2 to the M phase (18, 55). While previous studies have delineated AURKA’s involvement in HCC (56–58) and PMF (25, 59), its role in liver fibrosis remains underexplored. This study demonstrates that AURKA expression is elevated in activated HSCs and liver fibrosis. Furthermore, targeting AURKA with MLN8237 has shown promising results in reducing liver fibrosis and HSC activation both *in vivo* and *in vitro*.

Previous research has demonstrated that the Wnt signaling pathway activates β-catenin during liver fibrosis and the activation of HSCs (60–65). Our current study utilized RNA sequencing, bioinformatics analysis, RT-qPCR, and Western blot assay to suggest that Wnt/β-catenin may serve as the downstream pathway of AURKA. Xia et al. found that AURKA competes with β-catenin for AXIN binding, disrupting the degradation complex composed of AXIN, GSK3β and β-catenin, leads to increased β-catenin protein stability and activation of the Wnt signaling pathway in glioma cells (66). Additionally, Dar AA et al. demonstrated that AURKA phosphorylates GSK3β at serine nine, which decreasing phosphorylated β-catenin levels while enhancing β-catenin expression and its nuclear translocation in gastric cancer cells (67). Furthermore, Yanze Yin et al. demonstrated that AURKA co-localizes and interacts with GSK-3β in the cytoplasm of hepatocytes. Inhibition of AURKA significantly reduced the level of β-catenin protein by lowering the phosphorylation of glycogen synthase kinase-3β (GSK-3β), thereby inhibiting liver regeneration (68). Our study found that silencing AURKA significantly inhibits Wnt6 mRNA expression, which acts as the upstream regulator of the Wnt/β-catenin pathway. Moreover, silencing AURKA suppresses GSK3β mRNA and protein expression, ultimately leading to a reduction in the expression of its downstream target, β-catenin. This cascade of events results in decreased transcription of Wnt/β-Catenin target genes such as TCF1, MMP7, and CCND1. However, the mechanism by which AURKA regulates the transcription of WNT6 and the transcription and protein levels of GSK3B, ultimately affecting the levels of β-catenin protein, remains unclear. Our results suggest that AURKA may inhibit the expression of Wnt6, the upstream regulator of the classical Wnt/β-catenin signaling pathway, which in turn may further suppress the expression of intracellular DVL. This suppression could lead to an increased expression of GSK-3β protein, thereby enhancing the role of the GSK-3β degradation complex in the degradation of β-catenin. These findings emphasize that AURKA may facilitate HSC activation and exacerbate liver fibrosis through the Wnt/β-catenin pathway, highlighting its potential as a therapeutic target for liver fibrosis with minimal side effects. Future research is necessary to further elucidate the role of AURKA in liver fibrosis induced by various etiologies, as well as to investigate the intricate regulatory relationship between AURKA and Wnt/β-catenin both *in vivo* and *in vitro*.

## Data Availability

The datasets presented in this study can be found in online repositories. The names of the repository/repositories and accession number(s) can be found in the article/**Supplementary Material**.
