# Supplementary material for: Aurora kinase A promotes hepatic stellate cell activation and liver fibrosis through the Wnt/β-catenin pathway
Source: Front Oncol. 2025 Jan 6;14:1517226. doi: 10.3389/fonc.2024.1517226 (PMC11743346; doi:10.3389/fonc.2024.1517226)
Supplement: Supplementary file 1 [file DataSheet1.docx]

**Figure S1.** Body weight of mice in different groups during four weeks treatment with MLN8237.


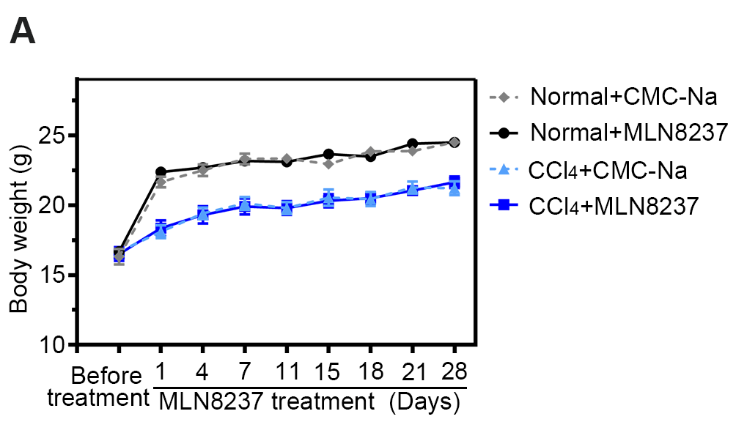


**Table S1.** Primary Antibodies for immunohistochemistry staining and western blot.

| Primary antibody | Manufacturer | Catalog  number | Dilution  factor |
| --- | --- | --- | --- |
| Aurora A | Biorbyt (Cambridge, UK) | Orb224015 | 100 |
| Aurora A | Abcam (Cambridge, MA) | Ab13824 | 100 |
| P-AURKA | CST (Danvers, MA) | 2914S | 500 |
| α-SMA | Abcam (Cambridge, MA) | ab7817 | 1000 |
| PDGFRβ | CST (Danvers, MA) | 3169T | 500 |
| β-catenin | Abcam (Cambridge, MA) | ab32572 | 10000 |
| GSK3β | Protein teach (Chicago, USA) | 22104-1-AP | 1000 |
| GAPDH | Bioword (Minnesota, USA) | AP0063 | 10000 |

**Table S2.** Real-time polymerase chain reaction primers.

| Gene | Primer sequence (5′ to 3′) | Predicted size |
| --- | --- | --- |
| AURKA | F: CCACCTTCGGCATCCTAATA | 91 bp |
|  | R: TCCAAGTGGTGCATATTCCA |  |
| ACTA2 | F: TTGGCTTGGCTTGTCAGG | 78 bp |
|  | R: GCTTTAGGGTCGCTGGAG |  |
| PDGFRβ | F: TTCCATGAGGATGCTGAGGT | 88 bp |
|  | R: CAGGGTGGCTCTCACTTAGC |  |
| TIMP1 | F: TGTTGTTGCTGTGGCTGATAGC | 118 bp |
|  | R: TCTGGTGTCCCCACGAACTT |  |
| MMP1 | F: CCAGAAGAACTGGTACATCAGCAA | 96 bp |
|  | R: CCGCCATACTCGAACTGGAA |  |
| PAI-1 | F: AGTGGACTTTTCAGAGGTGGA | 109 bp |
|  | R: GCCGTTGAAGTAGAGGGCAT |  |
| COL1A1 | F: CCAGAAGAACTGGTACATCAGCAA | 96 bp |
|  | R: CCGCCATACTCGAACTGGAA |  |
| CCND1 | F: ACAAACAGATCATCCGCAAACAC | 144 bp |
|  | R: TGTTGGGGCTCCTCAGGTTC |  |
| β-ACTIN | F: TGGCACCCAGCACAATGAA | 186 bp |
|  | R: CTAAGTCATAGTCCGCCTAGAAGCA |  |

**Table S3.** List of differentiated expressed genes after silencing AURKA expression in LX-2 cells.

| Gene_id | Gene_Symbol | log2FoldChange | *P* value |
| --- | --- | --- | --- |
| ENSG00000105877.17 | DNAH11 | 4.404397231 | 4.25E-14 |
| ENSG00000264235.5 | AP005329.1 | 4.357793412 | 1.39E-12 |
| ENSG00000261915.6 | AC026954.2 | 9.186598696 | 1.96E-11 |
| ENSG00000167774.2 | AC010323.1 | -9.395302558 | 3.03E-11 |
| ENSG00000256646.7 | AC010132.3 | 23.44714696 | 1.96E-09 |
| ENSG00000262481.5 | TMEM256-PLSCR3 | 4.329536284 | 4.31E-09 |
| ENSG00000254959.6 | INMT-MINDY4 | -22.20418461 | 1.35E-08 |
| ENSG00000261796.1 | ISY1-RAB43 | 22.14282522 | 1.46E-08 |
| ENSG00000265590.9 | AP000275.2 | -7.175197823 | 1.77E-08 |
| ENSG00000143882.10 | ATP6V1C2 | 3.816715469 | 5.99E-08 |
| ENSG00000249240.2 | AC069368.1 | 7.912831497 | 9.21E-08 |
| ENSG00000261771.5 | DNAAF4-CCPG1 | 20.82264926 | 9.99E-08 |
| ENSG00000151967.18 | SCHIP1 | 3.051199272 | 1.12E-07 |
| ENSG00000283149.1 | AC068631.2 | -7.898264759 | 1.24E-07 |
| ENSG00000230342.2 | FANCD2P2 | 20.35613455 | 1.92E-07 |
| ENSG00000276612.3 | FP565260.2 | 20.12034622 | 2.63E-07 |
| ENSG00000256861.1 | AC048338.1 | -4.281398939 | 3.88E-07 |
| ENSG00000076067.12 | RBMS2 | 2.085898231 | 5.18E-07 |
| ENSG00000273046.1 | AC012531.2 | -7.344033716 | 2.03E-05 |
| ENSG00000257315.2 | ZBED6 | -2.311192542 | 6.36E-05 |
| ENSG00000245662.3 | LINC02211 | 7.350202533 | 7.79E-05 |
| ENSG00000283897.1 | AC011416.4 | 6.645570917 | 8.02E-05 |
| ENSG00000131115.15 | ZNF227 | 1.690267967 | 0.0001051 |
| ENSG00000257390.5 | AC023055.1 | 2.718605227 | 0.0001193 |
| ENSG00000142973.13 | CYP4B1 | 6.757127841 | 0.0001201 |
| ENSG00000280417.1 | AC096887.2 | 2.34464082 | 0.0001549 |
| ENSG00000196810.4 | CTBP1-AS2 | -1.654325038 | 0.0002244 |
| ENSG00000113356.11 | POLR3G | -1.691075943 | 0.0002897 |
| ENSG00000139146.13 | FAM60A | -1.486671953 | 0.000292 |
| ENSG00000171365.15 | CLCN5 | -2.567167052 | 0.0003774 |
| ENSG00000119888.10 | EPCAM | 6.319839003 | 0.0004276 |
| ENSG00000179818.13 | PCBP1-AS1 | -1.526621715 | 0.0004331 |
| ENSG00000189367.14 | KIAA0408 | -2.244940418 | 0.0004898 |
| ENSG00000145217.13 | SLC26A1 | 1.489096819 | 0.000543 |
| ENSG00000184574.9 | LPAR5 | -6.385437229 | 0.0005728 |
| ENSG00000260643.2 | AC092718.3 | 6.717298124 | 0.0005854 |
| ENSG00000087586.17 | AURKA | -1.283131502 | 0.0006634 |
| ENSG00000284118.1 | MIR4707 | -5.501289281 | 0.0008286 |
| ENSG00000273049.1 | AC012531.3 | 5.593638364 | 0.0008639 |
| ENSG00000171466.9 | ZNF562 | -1.71939409 | 0.0008854 |
| ENSG00000258465.6 | AL139011.2 | -4.529078027 | 0.0008989 |
| ENSG00000162735.18 | PEX19 | -1.331743852 | 0.0009189 |
| ENSG00000058091.16 | CDK14 | 1.660795988 | 0.001129 |
| ENSG00000164466.12 | SFXN1 | -1.181363165 | 0.0011963 |
| ENSG00000248710.1 | AC079594.2 | 2.901599808 | 0.0012749 |
| ENSG00000198326.9 | TMEM239 | -6.300981672 | 0.0012892 |
| ENSG00000232456.1 | AL355994.2 | -6.809650121 | 0.001339 |
| ENSG00000257767.2 | AC002996.1 | -6.71106216 | 0.0015952 |
| ENSG00000198794.11 | SCAMP5 | -1.78062416 | 0.0017091 |
| ENSG00000158985.13 | CDC42SE2 | -1.176147542 | 0.0017154 |
| ENSG00000284671.1 | AC009093.9 | -4.899778741 | 0.0017422 |
| ENSG00000273333.2 | AL662884.1 | 7.343657461 | 0.0018328 |
| ENSG00000201129.1 | SNORA58B | -3.556851563 | 0.0019062 |
| ENSG00000284526.1 | AC015802.6 | -1.74301782 | 0.0021149 |
| ENSG00000198774.4 | RASSF9 | 5.807309754 | 0.0021898 |
| ENSG00000198142.4 | SOWAHC | -1.350628968 | 0.0023021 |
| ENSG00000244462.7 | RBM12 | -1.275172696 | 0.0023584 |
| ENSG00000277734.7 | TRAC | 5.888278611 | 0.0024274 |
| ENSG00000135272.10 | MDFIC | -1.989822773 | 0.0024935 |
| ENSG00000256249.1 | AC026333.3 | 5.785715305 | 0.0025748 |
| ENSG00000258790.1 | AL121594.3 | 1.84591596 | 0.0026358 |
| ENSG00000284740.1 | AL645728.2 | 5.957572855 | 0.0026716 |
| ENSG00000188582.8 | PAQR9 | 5.769097452 | 0.002738 |
| ENSG00000145949.9 | MYLK4 | 5.776545564 | 0.0028588 |
| ENSG00000269053.1 | AC010319.3 | -4.979941766 | 0.0028684 |
| ENSG00000273291.5 | AC092042.3 | 2.45749976 | 0.0030053 |
| ENSG00000254870.5 | ATP6V1G2-DDX39B | -9.71153842 | 0.0031396 |
| ENSG00000086827.8 | ZW10 | 1.217459366 | 0.0032346 |
| ENSG00000213397.10 | HAUS7 | 1.140529813 | 0.0033496 |
| ENSG00000264058.1 | AC073508.2 | 1.331415193 | 0.003361 |
| ENSG00000268790.5 | AC008764.4 | 4.850880404 | 0.0034017 |
| ENSG00000205930.8 | C21orf62-AS1 | 5.687230491 | 0.003574 |
| ENSG00000276077.4 | CU633904.1 | 6.301608742 | 0.00365 |
| ENSG00000112062.10 | MAPK14 | -1.565328414 | 0.0036608 |
| ENSG00000119471.14 | HSDL2 | 1.07575883 | 0.0041317 |
| ENSG00000161640.15 | SIGLEC11 | -4.890078916 | 0.0041653 |
| ENSG00000255526.6 | NEDD8-MDP1 | 2.737101089 | 0.004205 |
| ENSG00000284413.2 | BTBD8 | -6.483140947 | 0.0042904 |
| ENSG00000248751.6 | AC004997.1 | -2.496273002 | 0.0043272 |
| ENSG00000164591.13 | MYOZ3 | -2.377943336 | 0.004682 |
| ENSG00000115596.3 | WNT6 | -5.911849025 | 0.0048024 |
| ENSG00000283154.2 | IQCJ-SCHIP1 | -1.262787706 | 0.0049224 |
| ENSG00000092051.16 | JPH4 | -1.539919085 | 0.0052017 |
| ENSG00000130822.15 | PNCK | -4.766577657 | 0.0055189 |
| ENSG00000254486.1 | LINC02547 | -5.902140346 | 0.0056206 |
| ENSG00000215883.10 | CYB5RL | 1.253134345 | 0.0058359 |
| ENSG00000162073.13 | PAQR4 | 1.305220461 | 0.0059022 |
| ENSG00000038945.14 | MSR1 | 5.628589959 | 0.0061898 |
| ENSG00000128271.21 | ADORA2A | 2.078566129 | 0.0062745 |
| ENSG00000129473.9 | BCL2L2 | 1.535508717 | 0.0063232 |
| ENSG00000125999.10 | BPIFB1 | -6.277774763 | 0.0063477 |
| ENSG00000254099.1 | AC005740.3 | -5.952228495 | 0.0064566 |
| ENSG00000269113.3 | TRABD2B | -2.645580164 | 0.0067695 |
| ENSG00000234912.11 | SNHG20 | 1.767465175 | 0.0067857 |
| ENSG00000259120.2 | SMIM6 | -5.734918073 | 0.0069278 |
| ENSG00000254553.1 | AL033529.1 | -6.73979071 | 0.006933 |
| ENSG00000260108.1 | AC026464.2 | 3.352074976 | 0.0071433 |
| ENSG00000274512.5 | TBC1D3L | -1.485715889 | 0.0073915 |
| ENSG00000242265.5 | PEG10 | 1.513469815 | 0.0078103 |
| ENSG00000213625.8 | LEPROT | -1.791475298 | 0.0081539 |
| ENSG00000197965.11 | MPZL1 | -1.613731641 | 0.0085208 |
| ENSG00000112309.10 | B3GAT2 | -2.294436418 | 0.008547 |
| ENSG00000283900.1 | Z98749.3 | 5.148801638 | 0.0085718 |
| ENSG00000153561.12 | RMND5A | -1.287405084 | 0.0085757 |
| ENSG00000161920.9 | MED11 | 1.329407145 | 0.0086347 |
| ENSG00000082074.15 | FYB1 | 5.465010163 | 0.0086716 |
| ENSG00000110042.7 | DTX4 | -1.310641862 | 0.0087543 |
| ENSG00000267303.1 | AC011511.4 | 7.338880549 | 0.0088186 |
| ENSG00000262304.2 | AC027796.3 | -6.58963944 | 0.0088969 |
| ENSG00000188687.17 | SLC4A5 | 3.104222982 | 0.0090676 |
| ENSG00000060982.14 | BCAT1 | -1.086142429 | 0.0091086 |
| ENSG00000130545.15 | CRB3 | 5.382474302 | 0.009165 |
| ENSG00000267216.1 | AC020915.2 | -2.159268895 | 0.0096285 |
| ENSG00000184903.9 | IMMP2L | 1.605645559 | 0.0097552 |
| ENSG00000232729.7 | AC211433.1 | 2.911191451 | 0.0098345 |
| ENSG00000226520.1 | KIRREL1-IT1 | -5.670926363 | 0.0098847 |
| ENSG00000233013.10 | FAM157B | 5.804012996 | 0.0099742 |
| ENSG00000236432.7 | AC097662.1 | -2.584322153 | 0.010026 |
| ENSG00000271699.5 | SNX29P2 | 5.826741385 | 0.0100746 |
| ENSG00000274349.4 | ZNF658 | 1.369575234 | 0.010142 |
| ENSG00000267255.1 | AC011498.3 | -6.046157053 | 0.0101636 |
| ENSG00000227039.6 | ITGB2-AS1 | 5.370836617 | 0.0103181 |
| ENSG00000152455.15 | SUV39H2 | 1.051597923 | 0.0103434 |
| ENSG00000256349.1 | AP002748.4 | -10.01497774 | 0.0103754 |
| ENSG00000258945.1 | AL049775.3 | 5.400102754 | 0.0104594 |
| ENSG00000198894.7 | CIPC | 1.404789049 | 0.0105708 |
| ENSG00000272993.1 | AC239868.4 | 2.30350307 | 0.010712 |
| ENSG00000153094.22 | BCL2L11 | -1.953062456 | 0.0107295 |
| ENSG00000117758.13 | STX12 | 1.06418496 | 0.0108515 |
| ENSG00000092529.23 | CAPN3 | 4.042441593 | 0.0113594 |
| ENSG00000198930.12 | CSAG1 | -3.983860918 | 0.0114301 |
| ENSG00000143570.17 | SLC39A1 | 1.352210611 | 0.0114966 |
| ENSG00000142784.15 | WDTC1 | -1.070983832 | 0.0115491 |
| ENSG00000236120.6 | AC110995.1 | 1.216243779 | 0.0116076 |
| ENSG00000109667.11 | SLC2A9 | -1.59591977 | 0.0118422 |
| ENSG00000157765.11 | SLC34A2 | 3.09950418 | 0.0118595 |
| ENSG00000275527.1 | AC100835.2 | 3.091058935 | 0.0118657 |
| ENSG00000229474.6 | PATL2 | 5.323305301 | 0.0119953 |
| ENSG00000140931.19 | CMTM3 | -1.370041399 | 0.0120604 |
| ENSG00000147576.15 | ADHFE1 | 2.523439299 | 0.0121296 |
| ENSG00000260482.3 | AC008870.1 | 6.268942381 | 0.0124034 |
| ENSG00000269487.1 | AC008635.1 | 5.745301463 | 0.0124416 |
| ENSG00000267567.1 | AC008736.2 | 5.2721337 | 0.0127128 |
| ENSG00000180096.11 | 1-Sep | -1.587131702 | 0.0127288 |
| ENSG00000168907.13 | PLA2G4F | 5.796802537 | 0.012767 |
| ENSG00000134769.21 | DTNA | 2.216407702 | 0.0128766 |
| ENSG00000217576.7 | AL158066.1 | -6.41589302 | 0.0129433 |
| ENSG00000166159.10 | LRTM2 | 5.257193869 | 0.0130819 |
| ENSG00000166106.3 | ADAMTS15 | -1.036624851 | 0.0132069 |
| ENSG00000135373.12 | EHF | 5.327715418 | 0.0133364 |
| ENSG00000261757.1 | AC005592.1 | 5.248685042 | 0.0134991 |
| ENSG00000249661.1 | TNRC18P1 | 5.35037044 | 0.0136186 |
| ENSG00000169891.17 | REPS2 | -1.438536324 | 0.0137162 |
| ENSG00000264801.1 | ERVFRD-3 | -5.756769094 | 0.0137323 |
| ENSG00000241985.1 | WWTR1-IT1 | -5.59874868 | 0.0138583 |
| ENSG00000119782.13 | FKBP1B | -1.300486181 | 0.0139586 |
| ENSG00000283936.1 | MIR3658 | 4.238109735 | 0.0142228 |
| ENSG00000284336.1 | MIR2277 | -4.016487395 | 0.0142532 |
| ENSG00000128510.10 | CPA4 | -1.459880484 | 0.0142929 |
| ENSG00000197385.5 | ZNF860 | 3.58977476 | 0.0143223 |
| ENSG00000223969.5 | AC002456.1 | 5.687127351 | 0.0143337 |
| ENSG00000058404.19 | CAMK2B | -5.867925177 | 0.0143533 |
| ENSG00000273920.1 | AC103858.2 | 4.154403346 | 0.0145062 |
| ENSG00000265118.5 | AC134669.2 | 5.64743113 | 0.0146263 |
| ENSG00000148730.6 | EIF4EBP2 | -1.097521582 | 0.0146911 |
| ENSG00000279791.1 | AC018892.3 | 5.238399477 | 0.0148862 |
| ENSG00000280893.1 | AC009133.6 | -6.340067719 | 0.0151552 |
| ENSG00000257242.7 | LINC01619 | -2.929402662 | 0.0151752 |
| ENSG00000283029.1 | AL139099.5 | -9.465243913 | 0.0154166 |
| ENSG00000159337.6 | PLA2G4D | -5.875173282 | 0.0156018 |
| ENSG00000264812.1 | AC132938.2 | -5.180534645 | 0.0156658 |
| ENSG00000111540.15 | RAB5B | -1.713895018 | 0.0159249 |
| ENSG00000259753.1 | AC068234.1 | 6.145083772 | 0.016331 |
| ENSG00000171116.7 | HSFX1 | 6.165091706 | 0.0163726 |
| ENSG00000152580.8 | IGSF10 | 2.165682717 | 0.016451 |
| ENSG00000070731.10 | ST6GALNAC2 | 2.082466754 | 0.0165813 |
| ENSG00000145244.11 | CORIN | 5.214098509 | 0.0166392 |
| ENSG00000167562.12 | ZNF701 | -1.457589699 | 0.0166986 |
| ENSG00000272143.1 | FGF14-AS2 | 1.567368649 | 0.0167077 |
| ENSG00000163428.3 | LRRC58 | -1.311590179 | 0.0167755 |
| ENSG00000253943.1 | KRT18P37 | -5.78703157 | 0.0168479 |
| ENSG00000219027.2 | RPS3AP2 | 5.203216538 | 0.0168852 |
| ENSG00000179583.18 | CIITA | 1.462708805 | 0.0169434 |
| ENSG00000037241.7 | RPL26L1 | 1.073038175 | 0.0170527 |
| ENSG00000251580.1 | LINC02482 | 2.242747267 | 0.0171241 |
| ENSG00000276070.4 | CCL4L2 | -5.431968333 | 0.0171328 |
| ENSG00000165061.14 | ZMAT4 | 5.266299867 | 0.0171399 |
| ENSG00000246763.6 | RGMB-AS1 | 1.32852207 | 0.0171424 |
| ENSG00000263050.1 | AC090617.6 | -2.351522243 | 0.0174028 |
| ENSG00000072736.18 | NFATC3 | -1.240274848 | 0.0176292 |
| ENSG00000255073.8 | ZFP91-CNTF | -6.243348095 | 0.0180559 |
| ENSG00000178252.17 | WDR6 | -1.167103484 | 0.0180969 |
| ENSG00000270696.1 | AC005034.3 | -1.083451757 | 0.0181929 |
| ENSG00000275198.1 | AL512791.2 | 5.172813456 | 0.0184351 |
| ENSG00000232564.3 | AL031594.1 | 5.583041968 | 0.0184455 |
| ENSG00000158296.13 | SLC13A3 | 1.599634443 | 0.0184505 |
| ENSG00000157766.15 | ACAN | -5.827654995 | 0.0185379 |
| ENSG00000283662.1 | AC138904.3 | -5.827654995 | 0.0185379 |
| ENSG00000201675.1 | SNORD32A | -3.84577043 | 0.0191045 |
| ENSG00000246283.2 | AC090510.1 | 1.493543282 | 0.0192517 |
| ENSG00000155511.17 | GRIA1 | 5.602772621 | 0.0192625 |
| ENSG00000246851.1 | AL157938.3 | -5.792854918 | 0.0192887 |
| ENSG00000213443.2 | AC007068.1 | 2.065928419 | 0.0193164 |
| ENSG00000123268.8 | ATF1 | 1.068293742 | 0.0194133 |
| ENSG00000214894.6 | LINC00243 | 5.120207327 | 0.0195547 |
| ENSG00000124208.16 | TMEM189-UBE2V1 | -1.53716509 | 0.0195838 |
| ENSG00000148926.9 | ADM | -1.069744525 | 0.0197735 |
| ENSG00000171798.17 | KNDC1 | -1.295514125 | 0.0198359 |
| ENSG00000175267.14 | VWA3A | 5.140426282 | 0.0198697 |
| ENSG00000012211.12 | PRICKLE3 | -5.780807899 | 0.0199323 |
| ENSG00000127954.12 | STEAP4 | 5.217239359 | 0.0199615 |
| ENSG00000169871.12 | TRIM56 | 1.083974278 | 0.0200124 |
| ENSG00000111186.12 | WNT5B | 1.002369319 | 0.02003 |
| ENSG00000176402.5 | GJC3 | -5.379159831 | 0.0203251 |
| ENSG00000068781.21 | STON1-GTF2A1L | 2.551548908 | 0.0207697 |
| ENSG00000260095.1 | AC106820.3 | 2.367760817 | 0.0208333 |
| ENSG00000204611.6 | ZNF616 | 1.105041682 | 0.0209953 |
| ENSG00000164898.12 | FMC1 | 1.124317259 | 0.0209982 |
| ENSG00000229922.5 | LINC02528 | -5.336938686 | 0.0210125 |
| ENSG00000231738.10 | TSPAN19 | 5.112685661 | 0.0211705 |
| ENSG00000163501.6 | IHH | 3.7099101 | 0.0212551 |
| ENSG00000092607.13 | TBX15 | 5.126986377 | 0.0213802 |
| ENSG00000249962.1 | AL157400.5 | 1.973343018 | 0.0214618 |
| ENSG00000267059.2 | AC005943.1 | -1.935993475 | 0.0218951 |
| ENSG00000141524.15 | TMC6 | -1.356847176 | 0.022015 |
| ENSG00000155749.12 | ALS2CR12 | 3.190224776 | 0.0220278 |
| ENSG00000269955.2 | C7orf55-LUC7L2 | 8.943375359 | 0.0220926 |
| ENSG00000170743.16 | SYT9 | -5.324980435 | 0.0221261 |
| ENSG00000258555.6 | SPECC1L-ADORA2A | -1.700899626 | 0.0222814 |
| ENSG00000165832.5 | TRUB1 | -1.250053471 | 0.0223101 |
| ENSG00000196950.13 | SLC39A10 | -1.368078161 | 0.0223604 |
| ENSG00000120051.14 | CFAP58 | 1.487928709 | 0.0228354 |
| ENSG00000139304.12 | PTPRQ | 2.244844563 | 0.0228458 |
| ENSG00000157653.11 | C9orf43 | 1.159963475 | 0.0229832 |
| ENSG00000185303.16 | SFTPA2 | 2.196888352 | 0.0231604 |
| ENSG00000113742.12 | CPEB4 | -1.453480189 | 0.0232207 |
| ENSG00000235750.9 | KIAA0040 | 5.977039731 | 0.023293 |
| ENSG00000267561.2 | AC093155.3 | 5.050707715 | 0.0233582 |
| ENSG00000139579.12 | NABP2 | 1.020978748 | 0.0233663 |
| ENSG00000110092.3 | CCND1 | -1.147950646 | 0.0236193 |
| ENSG00000196227.10 | FAM217B | -1.154674842 | 0.0237825 |
| ENSG00000226948.1 | RPS4XP2 | 5.497715351 | 0.0240466 |
| ENSG00000253455.1 | AC022915.1 | 5.07051663 | 0.0241228 |
| ENSG00000259080.1 | AC074091.1 | 2.531743966 | 0.0242289 |
| ENSG00000175591.11 | P2RY2 | -2.684529938 | 0.0243026 |
| ENSG00000229619.3 | MBNL1-AS1 | 1.156039925 | 0.0244267 |
| ENSG00000058673.16 | ZC3H11A | 3.243586694 | 0.024445 |
| ENSG00000270604.5 | HCG17 | 5.039021568 | 0.0246844 |
| ENSG00000215915.9 | ATAD3C | 5.918412814 | 0.0250405 |
| ENSG00000100360.14 | IFT27 | 1.001770386 | 0.0250515 |
| ENSG00000144063.3 | MALL | 5.43210194 | 0.0251249 |
| ENSG00000253311.2 | LINC01847 | -5.618963438 | 0.0251401 |
| ENSG00000114771.13 | AADAC | 5.062882314 | 0.0251789 |
| ENSG00000244267.1 | RPL34P22 | -5.676047273 | 0.0254865 |
| ENSG00000005102.12 | MEOX1 | -5.264010197 | 0.025536 |
| ENSG00000100014.19 | SPECC1L | -1.055980592 | 0.0258702 |
| ENSG00000137709.9 | POU2F3 | -3.873168813 | 0.0258893 |
| ENSG00000132329.10 | RAMP1 | -5.679937227 | 0.0259578 |
| ENSG00000230433.1 | AL031770.1 | -5.679937227 | 0.0259578 |
| ENSG00000280278.1 | FLJ30679 | 5.437968292 | 0.0259729 |
| ENSG00000280078.1 | AC016526.3 | -5.256094548 | 0.026045 |
| ENSG00000267179.1 | AC008770.3 | 3.93365761 | 0.0261051 |
| ENSG00000111181.12 | SLC6A12 | -5.631198771 | 0.026139 |
| ENSG00000165806.19 | CASP7 | 1.385574828 | 0.0262741 |
| ENSG00000143450.16 | OAZ3 | -1.19767648 | 0.0263503 |
| ENSG00000164692.17 | COL1A2 | 1.051602694 | 0.0265144 |
| ENSG00000205002.3 | AARD | 5.009285822 | 0.0267815 |
| ENSG00000125046.14 | SSUH2 | 1.575055457 | 0.0272932 |
| ENSG00000244198.5 | AC004889.1 | -4.305858152 | 0.0275183 |
| ENSG00000262633.2 | AC005670.2 | -8.609562229 | 0.0275874 |
| ENSG00000166670.9 | MMP10 | -5.247012247 | 0.0277976 |
| ENSG00000185015.7 | CA13 | -1.637049299 | 0.028064 |
| ENSG00000183715.13 | OPCML | 1.256826459 | 0.0281343 |
| ENSG00000161055.3 | SCGB3A1 | -3.296892502 | 0.0281843 |
| ENSG00000203804.4 | ADAMTSL4-AS1 | -1.038766074 | 0.028221 |
| ENSG00000142959.4 | BEST4 | 5.00173994 | 0.0282275 |
| ENSG00000277117.4 | FP565260.3 | -1.19566846 | 0.0282969 |
| ENSG00000235018.1 | AL137077.1 | -5.214852777 | 0.0284924 |
| ENSG00000233093.5 | LINC00892 | 4.968225504 | 0.0289138 |
| ENSG00000079385.21 | CEACAM1 | -1.932294876 | 0.028986 |
| ENSG00000114786.16 | ABHD14A-ACY1 | 2.006667457 | 0.0290969 |
| ENSG00000257740.1 | AC073896.3 | -2.143671989 | 0.0291908 |
| ENSG00000043591.5 | ADRB1 | -1.929457439 | 0.0295539 |
| ENSG00000258643.5 | BCL2L2-PABPN1 | -1.180052075 | 0.0296643 |
| ENSG00000227467.3 | LINC01537 | 1.228295486 | 0.0296918 |
| ENSG00000254980.1 | AP002008.1 | -5.613505648 | 0.029718 |
| ENSG00000137699.16 | TRIM29 | -1.94754213 | 0.0300864 |
| ENSG00000100101.15 | Z83844.1 | -8.45922854 | 0.0304493 |
| ENSG00000234478.1 | ACBD3-AS1 | -4.064483005 | 0.0306184 |
| ENSG00000267047.1 | AC040977.2 | 8.446563056 | 0.0306597 |
| ENSG00000002079.14 | MYH16 | 1.21521646 | 0.0308038 |
| ENSG00000169155.9 | ZBTB43 | -1.399429593 | 0.030828 |
| ENSG00000166961.14 | MS4A15 | 5.413205426 | 0.0309689 |
| ENSG00000233626.2 | AC092814.1 | 4.362869203 | 0.0310028 |
| ENSG00000143850.13 | PLEKHA6 | -1.850202971 | 0.0311551 |
| ENSG00000276058.1 | STMN1P1 | 4.93408156 | 0.031175 |
| ENSG00000251018.2 | HMMR-AS1 | -4.265517559 | 0.0314188 |
| ENSG00000186272.12 | ZNF17 | 1.263890214 | 0.0316036 |
| ENSG00000100068.11 | LRP5L | -1.041026503 | 0.0316661 |
| ENSG00000100311.16 | PDGFB | -1.089718674 | 0.0317639 |
| ENSG00000250132.6 | AC004803.1 | 1.006614193 | 0.0319374 |
| ENSG00000121316.10 | PLBD1 | 5.365729761 | 0.0319987 |
| ENSG00000216990.2 | HSPD1P10 | 4.942491361 | 0.032013 |
| ENSG00000277142.1 | Z98882.1 | 1.216648555 | 0.0320682 |
| ENSG00000258940.2 | AL132639.2 | 1.893409064 | 0.0321989 |
| ENSG00000131797.12 | CLUHP3 | -1.136070102 | 0.032543 |
| ENSG00000248405.10 | PRR5-ARHGAP8 | 4.923280133 | 0.0330376 |
| ENSG00000283178.1 | BX119927.1 | -5.521229341 | 0.0332855 |
| ENSG00000166197.16 | NOLC1 | 1.061119945 | 0.0336093 |
| ENSG00000144596.12 | GRIP2 | -1.455154268 | 0.0339211 |
| ENSG00000133110.14 | POSTN | 3.713834219 | 0.0342631 |
| ENSG00000278926.1 | AC092134.2 | -5.145360932 | 0.0347138 |
| ENSG00000240211.1 | AC092849.1 | 5.012303845 | 0.0348333 |
| ENSG00000236393.1 | AC091806.1 | -1.375802577 | 0.0349063 |
| ENSG00000284735.1 | AL139424.3 | 4.918053406 | 0.034974 |
| ENSG00000106483.11 | SFRP4 | 5.291769548 | 0.0350215 |
| ENSG00000267605.5 | AC016590.1 | -3.886697729 | 0.0350911 |
| ENSG00000010319.6 | SEMA3G | 5.328183928 | 0.035122 |
| ENSG00000267505.1 | AC005180.2 | 2.236039403 | 0.0351644 |
| ENSG00000167131.16 | CCDC103 | -1.464406195 | 0.0352688 |
| ENSG00000214872.8 | SMTNL1 | -3.28084088 | 0.0353834 |
| ENSG00000090857.13 | PDPR | -1.413484794 | 0.0353916 |
| ENSG00000166796.11 | LDHC | 4.882986446 | 0.0356024 |
| ENSG00000259529.1 | AL136295.5 | -1.411692086 | 0.035927 |
| ENSG00000144668.11 | ITGA9 | 2.058237332 | 0.035998 |
| ENSG00000182057.4 | OGFRP1 | 2.167499815 | 0.0361995 |
| ENSG00000183479.12 | TREX2 | -1.469902183 | 0.036261 |
| ENSG00000267121.5 | AC008105.3 | -1.600017269 | 0.0362966 |
| ENSG00000196284.15 | SUPT3H | 1.025604973 | 0.036345 |
| ENSG00000234753.5 | FOXP4-AS1 | 4.869633741 | 0.0364346 |
| ENSG00000164938.13 | TP53INP1 | -1.419618671 | 0.0370363 |
| ENSG00000151553.14 | FAM160B1 | -1.230197322 | 0.037275 |
| ENSG00000243491.1 | AC082651.3 | 5.252180036 | 0.0374008 |
| ENSG00000234695.1 | AC002076.1 | 4.899993962 | 0.0374652 |
| ENSG00000179902.12 | C1orf194 | 4.878121424 | 0.0374984 |
| ENSG00000198155.5 | ZNF876P | 3.268965532 | 0.0375916 |
| ENSG00000172771.11 | EFCAB12 | 2.596135633 | 0.0376279 |
| ENSG00000165383.11 | LRRC18 | -5.877592278 | 0.0380566 |
| ENSG00000263715.7 | LINC02210-CRHR1 | 4.835962414 | 0.038068 |
| ENSG00000066468.22 | FGFR2 | 5.252172512 | 0.0381613 |
| ENSG00000261147.1 | AC091167.3 | -3.236619692 | 0.0381777 |
| ENSG00000125898.12 | FAM110A | -1.28307977 | 0.0383158 |
| ENSG00000231856.2 | AL162377.1 | 2.042881978 | 0.038359 |
| ENSG00000180776.15 | ZDHHC20 | -1.42189913 | 0.0383785 |
| ENSG00000248636.6 | AC002070.1 | -1.095888866 | 0.0385295 |
| ENSG00000271949.1 | AC093423.3 | -5.406248409 | 0.038754 |
| ENSG00000230673.3 | PABPC1P3 | 3.204101251 | 0.0389633 |
| ENSG00000111254.7 | AKAP3 | 1.404699535 | 0.0389638 |
| ENSG00000204044.6 | SLC12A5-AS1 | -1.352015923 | 0.0390332 |
| ENSG00000127743.5 | IL17B | 4.847489848 | 0.0390489 |
| ENSG00000273027.1 | AL844908.2 | 4.858714401 | 0.0390617 |
| ENSG00000235554.1 | AC005822.1 | -3.561270163 | 0.0391663 |
| ENSG00000228175.3 | GEMIN8P4 | 1.762581397 | 0.0393141 |
| ENSG00000261678.2 | SCRT1 | 4.869863498 | 0.0394332 |
| ENSG00000232160.6 | RAP2C-AS1 | 1.717282903 | 0.0394868 |
| ENSG00000151576.10 | QTRT2 | -1.127539124 | 0.0397847 |
| ENSG00000235042.1 | AC098820.2 | 4.824792936 | 0.0398437 |
| ENSG00000230417.11 | LINC00595 | 1.395435384 | 0.0400577 |
| ENSG00000267952.1 | AC008878.1 | 5.664375682 | 0.0400626 |
| ENSG00000204581.2 | ACOXL-AS1 | 4.813311794 | 0.0405836 |
| ENSG00000235863.3 | B3GALT4 | -5.38308547 | 0.0407942 |
| ENSG00000263644.1 | AC005828.3 | -5.38308547 | 0.0407942 |
| ENSG00000181634.7 | TNFSF15 | -2.058774972 | 0.0410575 |
| ENSG00000141574.7 | SECTM1 | -1.740877548 | 0.041729 |
| ENSG00000178977.3 | LINC00324 | 3.3099121 | 0.0417641 |
| ENSG00000178761.14 | FAM219B | -1.214702583 | 0.0419636 |
| ENSG00000198018.6 | ENTPD7 | -1.083248859 | 0.0423223 |
| ENSG00000242073.2 | AC211429.1 | 3.33365292 | 0.0423834 |
| ENSG00000182985.17 | CADM1 | 1.847701731 | 0.0425283 |
| ENSG00000139645.9 | ANKRD52 | -1.002631768 | 0.0430128 |
| ENSG00000255624.2 | AC073585.1 | 2.055445069 | 0.0430531 |
| ENSG00000184956.15 | MUC6 | 4.810103739 | 0.0434046 |
| ENSG00000258733.5 | LINC02328 | 2.116084132 | 0.043879 |
| ENSG00000167105.7 | TMEM92 | -3.893163946 | 0.043916 |
| ENSG00000158270.11 | COLEC12 | 2.923721233 | 0.0439941 |
| ENSG00000074047.21 | GLI2 | -1.044051285 | 0.0440102 |
| ENSG00000179195.15 | ZNF664 | 1.209292553 | 0.0442923 |
| ENSG00000250657.1 | AC097451.1 | 4.829903185 | 0.0446817 |
| ENSG00000188388.10 | GOLGA6L3 | 5.572215648 | 0.0447167 |
| ENSG00000139351.14 | SYCP3 | 2.040012647 | 0.0447975 |
| ENSG00000244295.2 | RPS20P21 | -2.536160231 | 0.0451469 |
| ENSG00000230869.1 | AGAP10P | 5.773580922 | 0.0453611 |
| ENSG00000136144.11 | RCBTB1 | -1.009445723 | 0.0455788 |
| ENSG00000267551.3 | AC005264.1 | 5.135148891 | 0.0457533 |
| ENSG00000235529.1 | AGAP1-IT1 | -1.991317704 | 0.0458775 |
| ENSG00000248027.1 | AP001351.1 | 3.122477363 | 0.0460236 |
| ENSG00000276470.1 | NPPA-AS1_1 | -3.225019965 | 0.0463513 |
| ENSG00000276384.1 | AC016876.3 | 1.806209728 | 0.046521 |
| ENSG00000249679.1 | AC106897.1 | -5.355497614 | 0.0468068 |
| ENSG00000277775.1 | HIST1H3F | -2.284420519 | 0.046832 |
| ENSG00000099994.10 | SUSD2 | 1.346308766 | 0.0470883 |
| ENSG00000258677.2 | AC022826.2 | 5.539851959 | 0.0471381 |
| ENSG00000081059.19 | TCF7 | -1.236429445 | 0.0475839 |
| ENSG00000248690.6 | HAS2-AS1 | 2.109442977 | 0.0477279 |
| ENSG00000273026.1 | AL358472.3 | 2.080359395 | 0.0480098 |
| ENSG00000112972.14 | HMGCS1 | 1.06350134 | 0.0480844 |
| ENSG00000065618.18 | COL17A1 | 1.070902126 | 0.0482507 |
| ENSG00000115226.9 | FNDC4 | 2.056585522 | 0.048285 |
| ENSG00000157782.9 | CABP1 | -1.801866569 | 0.0486333 |
| ENSG00000233178.7 | AL161457.2 | -1.790387223 | 0.0490798 |
| ENSG00000221995.5 | TIAF1 | 1.197416056 | 0.0492321 |
| ENSG00000205426.10 | KRT81 | -1.084640385 | 0.0492517 |
| ENSG00000254088.1 | SLC2A3P4 | 5.13527088 | 0.0492522 |
| ENSG00000276410.3 | HIST1H2BB | -3.882561163 | 0.0495376 |
| ENSG00000273173.5 | SNURF | -1.286868997 | 0.049875 |
| ENSG00000186765.11 | FSCN2 | -1.749986107 | 0.0499441 |

**Table S4.** Top 15 gene sets enriched in LX-2 cells treated with AURKA siRNA compared to the control group (n=3).

| GENE SETS | SIZE | NES | NOM p-val |
| --- | --- | --- | --- |
| KEGG_BLADDER_CANCER | 42 | -1.64 | 0.002 |
| KEGG_ACUTE_MYELOID_LEUKEMIA | 57 | -1.55 | 0.009 |
| KEGG_TYPE_II_DIABETES_MELLITUS | 47 | -1.54 | 0.007 |
| KEGG_CHRONIC_MYELOID_LEUKEMIA | 73 | -1.48 | 0.019 |
| KEGG_PANCREATIC_CANCER | 70 | -1.46 | 0.007 |
| KEGG_GLYCOSAMINOGLYCAN_BIOSYNTHESIS_HEPARAN_SULFATE | 26 | -1.42 | 0.040 |
| KEGG_INSULIN_SIGNALING_PATHWAY | 137 | -1.42 | 0.003 |
|  |  |  |  |
| KEGG_NATURAL_KILLER_CELL_MEDIATED_CYTOTOXICITY | 132 | -1.40 | 0.016 |
| KEGG_NON_SMALL_CELL_LUNG_CANCER | 54 | -1.36 | 0.038 |
| KEGG_ASCORBATE_AND_ALDARATE_METABOLISM | 25 | -1.35 | 0.074 |
| KEGG_JAK_STAT_SIGNALING_PATHWAY | 155 | -1.34 | 0.013 |
| KEGG_BASAL_CELL_CARCINOMA | 55 | -1.34 | 0.041 |
| **KEGG_WNT_SIGNALING_PATHWAY** | **150** | **-1.33** | **0.019** |
| KEGG_MELANOMA | 71 | -1.33 | 0.037 |
| KEGG_ERBB_SIGNALING_PATHWAY | 87 | -1.33 | 0.032 |
